# Increased expression of IRF8 in tumor cells inhibits the generation of Th17 cells and predicts unfavorable survival of diffuse large B cell lymphoma patients

**DOI:** 10.18632/oncotarget.17693

**Published:** 2017-05-08

**Authors:** Wei-Jie Zhong, Xin Xu, Zhi-Gang Zhu, Qing-Hua Du, Hong Du, Li Yang, Yan-Ying Ling, Hua-Bao Xiong, Qing-Shan Li

**Affiliations:** ^1^ Department of Hematology & Oncology, Guangzhou First People's Hospital, Guangzhou Medical University, Guangzhou, China; ^2^ Department of Hematology, Guangzhou First People's Hospital, Guangzhou Medical University, Guangzhou, China; ^3^ Immunology Institute, Mount Sinai School of Medicine, New York, NY, USA; ^4^ Department of Pathology, Guangzhou First People's Hospital, Guangzhou Medical University, Guangzhou, China; ^5^ Department of Laboratory, Guangzhou First People's Hospital, Guangzhou Medical University, Guangzhou, China; ^6^ Collaborative Innovation Center for Cancer Medicine, State Key Laboratory of Oncology in South China, Guangdong Esophageal Cancer Institute, Cancer Center, Sun Yat-Sen University, Guangzhou, China

**Keywords:** Interferon regulatory factor 8, Th17 cells, lymphoma, large B cell, diffuse

## Abstract

The immunological pathogenesis of diffuse large B cell lymphoma (DLBCL) remains elusive. Searching for new prognostic markers of DLBCL is a crucial focal point for clinical scientists. The aim of the present study was to examine the prognostic value of interferon regulatory factor 8 (IRF8) expression and its effect on the development of Th17 cells in the tumor microenvironment of DLBCL patients. Flow cytometry, immunohistochemistry, and quantitative real-time PCR were used to detect the distribution of Th17 cells and related cytokines and IRF8 in tumor tissues from DLBCL patients. Two DLBCL cell lines (OCI-LY10 and OCI-LY1) with IRF8 knockdown or overexpression and two human B lymphoblast cell lines were co-cultured with peripheral blood mononuclear cells (PBMCs) *in vitro* to determine the effect of IRF8 on the generation of Th17 cells. Quantitative real-time PCR and Western blotting were used to investigate the involvement of retinoic acid receptor-related orphan receptor gamma t (RORγt) in the effect of IRF8 on Th17 cell generation. The survival of 67 DLBCL patients was estimated using the Kaplan-Meier method and log-rank analysis. The percentage of Th17 cells was lower in DLBCL tumor tissues than in PBMCs and corresponding adjacent benign tissues. Relative expression of interleukin (IL)-17A was lower, whereas that of interferon (IFN)-γ was higher in tumor tissues than in benign tissues. Co-culture with DLBCL cell lines inhibited the generation of Th17 cells *in vitro*. IRF8 upregulation was detected in DLBCL tumor tissues, and it was associated with decreased DLBCL patient survival. Investigation of the underlying mechanism suggested that IRF8 upregulation in DLBCL, through an unknown mechanism, inhibited Th17 cell generation by suppressing RORγt in neighboring CD4+ T cells. Tumor cells may express soluble or membrane-bound factors that inhibit the expression of RORγt in T cells within the tumor microenvironment. Our findings suggest that IRF8 expression could be a prognostic factor for DLBCL.

## INTRODUCTION

Diffuse large B cell lymphoma (DLBCL) is the most common type of aggressive non-Hodgkin's lymphoma (NHL) and accounts for 30%–40% of NHLs in adults [1]. DLBCL consists of a group of malignancies with high heterogeneity in their clinical features, morphology, pathology, genetics, and prognosis [2, 3]. Although the first-line chemotherapy regimen R-CHOP (Rituximab, cyclophosphamide, doxorubicin, vincristine, and prednisone) has improved the prognosis of DLBCL patients in recent years [4, 5], many DLBCL patients are still refractory or relapsed. The International Prognostic Index (IPI) is a currently commonly used index to evaluate the clinical prognosis of DLBCL patients. However, many DLBCL patients with the same IPI scores have different prognoses, especially those with low IPI scores [6]. The IPI is mainly composed of clinical characteristics, and does not include the molecular pathology of DLBCL [6]. The pathogenesis of DLBCL, especially the immunological pathogenesis of the DLBCL microenvironment, remains elusive. Exploring the molecular mechanism and searching for new prognostic markers of DLBCL is an important focus topic for clinical scientists.

Th17 cells, a new subset of CD4^+^ T cells first identified in 2005 [7], secrete IL-17 and IL-21, and play a crucial role in autoimmune diseases [8]. Their differentiation is inhibited by transforming growth factor (TGF)-β; retinoid-related orphan receptor γt (ROR-γt) is one of the key transcription factors in Th17 cell differentiation [9–11]. Th17 cells, as a CD4+IL-17-producing subset of tumor-infiltrating lymphocytes (TILs), are found in the tumor microenvironment, including nasopharyngeal carcinoma, colon cancer, pancreatic cancer and ovarian cancer [12–15]. NHL is closely related to Th17 cells and associated cytokines, which are significantly lower in the peripheral blood from DLBCL patients [16–18]. In addition, the frequency of Th17 cells in peripheral blood significantly increased in relapsed patients [18]. However, the anti-tumor or pro-tumor role of Th17 cells in DLBCL remains controversial.

Interferon regulatory factor 8 (IRF8) plays important roles in hematopoietic cell development, survival and so on [19]. Studies suggest that IRF8 is involved in the tumorigenesis or progression of many tumors. Irf8^−/−^ mice develop a disorder similar to chronic myelogenous leukemia [20]. IRF8 accelerates the apoptosis of human colon carcinoma cells [21]. IRF8 also plays a role in B cell lymphoma. IRF8 is present at higher levels in germinal center (GC) derived lymphomas, whereas it is lowly expressed in marginal zone lymphomas and mantle cell lymphomas, especially in all GCB-type DLBCLs [22]. The gene fusion between immunoglobulin heavy chain and IRF8 leads to the IRF8 overexpression in tumor lesions [23]. A different study reported that IRF8 is associated with GCB-type DLBCL and involved in the (14;16)(q32.33;q24.1) chromosomal translocation, which indicates a poor prognosis [24]. Recently, it was demonstrated that loss of IRF8 inhibits the growth of DLBCL, and IRF8 may be an oncogenic factor in human DLBCL by suppressing the phosphorylation of p38 and ERK [25]. However, the role of IRF8 in DLBCL remains completely elusive.

The relationship between IRF8 and Th17 cell development in mice is controversial. Two recent studies showed that IRF8 suppressed the differentiation of Th17 cell [26, 27]. The increase in the propensity of IRF8-deficient T cells was correlated with colitis and uveitis [26, 27]. IRF8 might physically interact with RORγt and suppress the activity of IL-17 promoter [26]. By contrast, a study suggested that IRF8 promoted Th17 cell differentiation in mice [28]. However, whether the expression profile of IRF8 in human tumor cells affects the generation of Th17 cells and survival of DLBCL patients remains unknown.

In this research, we explored the distribution of Th17 cells, related cytokines, and IRF8 in patients with DLBCL. Our results showed that high levels of IRF8 in tumor cells inhibited the generation of Th17 cells and predicted unfavorable clinical outcomes in DLBCL patients.

## RESULTS

### The percentage of Th17 cells decreased in the DLBCL tumor microenvironment

**Figure 1:**
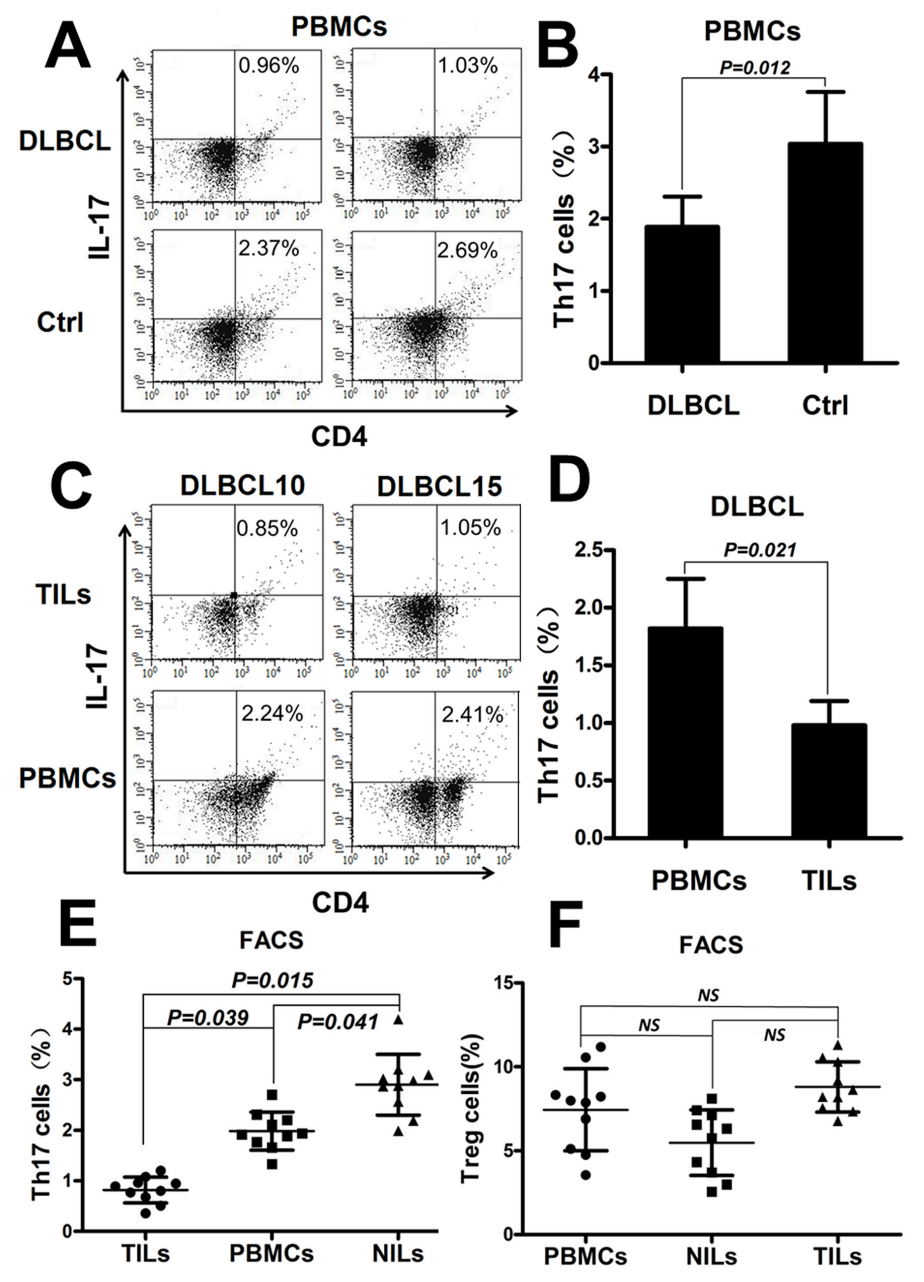
The percentage of Th17 cells decreased in peripheral blood and in the tumor microenvironment of DLBCL patients **(A)** Representative FACS plots of Th17 cells in the PBMCs from two DLBCL patients and two healthy volunteers (Ctrl). The number displayed is the percentage of Th17 cells. **(B)** Graph of the Th17 cell percentages in PBMCs from DLBCL patients (n=20) and Ctrl (n=20). **(C)** Representative FACS plots of Th17 cells in PBMCs and the tumor microenvironment from two matched DLBCL patients. **(D)** Graph of the Th17 cell mean percentages in PBMCs and TILs from DLBCL patients (n=20). **(E)** Graph of the Th17 cell percentages in matched sets of samples of PBMCs, tumor tissues (TILs), and corresponding adjacent benign tissues(NILs) from DLBCL patients (n=10). **(F)** Graph of the Treg cell percentages under the same conditions as in (E). Error bars represent standard deviation (SD). Significance was determined using independent-sample Student's t/t′ test (two groups) or single-factor analysis of variance (one-way ANOVA) Student-Newman-Keulor/Dunnett's T3 test (three groups). The *P* value is indicated in each graph.

We investigated the percentage of Th17 cells in peripheral blood mononuclear cells (PBMCs) and in TILs from 20 newly diagnosed DLBCL patients (Table 1) and 20 healthy volunteers. Representative fluorescence-activated cell sorting (FACS) plots of Th17 cells in the PBMCs of two DLBCL patients and two healthy volunteers are displayed in Figure 1A. The mean Th17 cells percentage was significantly lower in the PBMCs of DLBCL patients than in those of healthy volunteers (Figure 1B; *P* = 0.012). The percentage of Th17 cells was compared between tumor tissues and PBMCs by analyzing matched samples of 20 DLBCL patients. Representative FACS plots are shown in Figure 1C. The mean percentage of Th17 cells was significantly lower in tumor tissues than in PBMCs (Figure 1D; *P* = 0.021).

**Table 1:** Clinical characteristics of 20 DLBCL patients

Characteristics	No. (%)
**Age**	
Median	55
Range	32-76
**Gender**	
Male	12(60)
Female	8(40)
**Ann Arbor stage^a^**	
I-II	6(30)
III-IV	14(70)
**IPI score^b^**	
1-3	11(55)
4-5	9(45)

^a^Ann Arbor stage according to Ann Arbor-Cotswald stage (1989).

^b^IPI score, International Prognostic Index score.

To confirm that Th17 cells decreased in the DLBCL tumor microenvironment, we compared Th17 cells percentage in matched samples of PBMCs, tumor tissues and corresponding adjacent benign tissues from DLBCL patients (n = 10). The percentage of Th17 cells was significantly lower in tumor tissues than in PBMCs (*P* = 0.039) or adjacent tissues (*P* = 0.015) and lower in PBMCs than in adjacent tissues (*P* = 0.041) (Figure 1E). Regulatory T cells (Tregs) were also detected by FACS under the same conditions, and no statistically differences were detected among the three groups (Figure 1F).

### Expression of Th17 cell-related cytokines in tumor tissues and benign tissues

**Figure 2:**
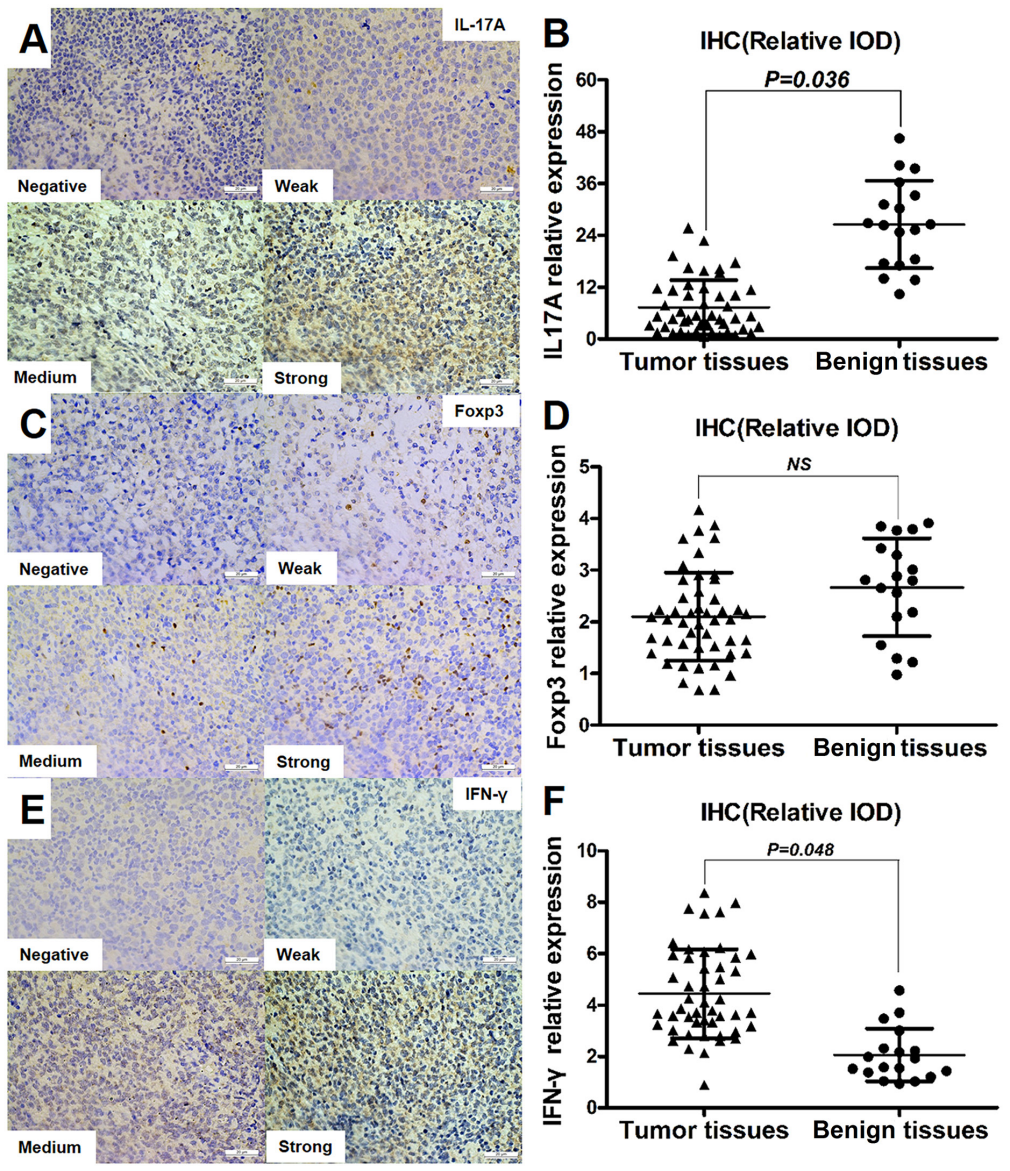
Expression of Th17 cell-related cytokines in tumor tissues and benign tissues Immunohistochemical staining showed various intensities of IL-17A **(A)**, FOXP3 **(C)**, and IFN-γ **(E)** expression in the cytoplasm or nucleus of tumor cells or TILs (×40). Graph of IL-17A **(B)**, FOXP3 **(D)**, and IFN-γ **(F)** relative expression (relative IOD) in IHCs from tumor tissues (n=48) and benign tissues (n=18). Error bars represent S.D. Significance was determined using independent-sample Student's t/t′ test. The *P* value is indicated in each graph.

To study the specific functions of Th17 cells in the DLBCL microenvironment, typical intracellular cytokines related to Th17cells, including IL-17A, IFN-γ, and FOXP3 were analyzed by immunohistochemistry (IHC) in paraffin-embedded tumor specimens from 48 DLBCL patients and benign lymph node specimens from 18 controls. The relative integrated optical density (IOD) was calculated from IHC images to represent the levels of cytokines. IL-17A and IFN-γ were expressed in the cytoplasm of tumor cells or TILs (Figure 2A and 2E), FOXP3 was expressed in the nucleus of tumor cells or TILs (Figure 2C). The relative expression of IL-17A was significantly lower in tumor tissues than in benign tissues (Figure 2B, *P* = 0.036). IFN-γ was expressed at higher levels in tumor tissues than in benign tissues (Figure 2F, *P* = 0.048). FOXP3 expression did not differ significantly between the two groups (Figure 2D).

### DLBCL cell lines inhibited the generation of Th17 cells and related cytokines *in vitro*

The mechanism underlying the reduced distribution of Th17 cells in the tumor microenvironment remains elusive. A previous research reported that tumor cells induce a decrease of Th17 cells in the B cell NHL microenvironment [17]. To determine whether DLBCL tumor cells affect Th17 cells generation, we firstly investigated the effect of DLBCL cells on the differentiation of Th17 cells from PBMCs *in vitro*. PBMCs from healthy donors were divided into six groups and co-cultured with the DLBCL cell lines OCI-LY10 (ABC subtype) and OCI-LY1 (GCB subtype), the human B lymphoblast cell lines WIL2S and DAKIKI at a 1:1 ratio, or in the presence of the cytokine TGF-β (3 ng/mL); the blank control group only included PBMCs. All six groups were co-cultured with IL-2 for 7 days and then detected by FACS.

**Figure 3:**
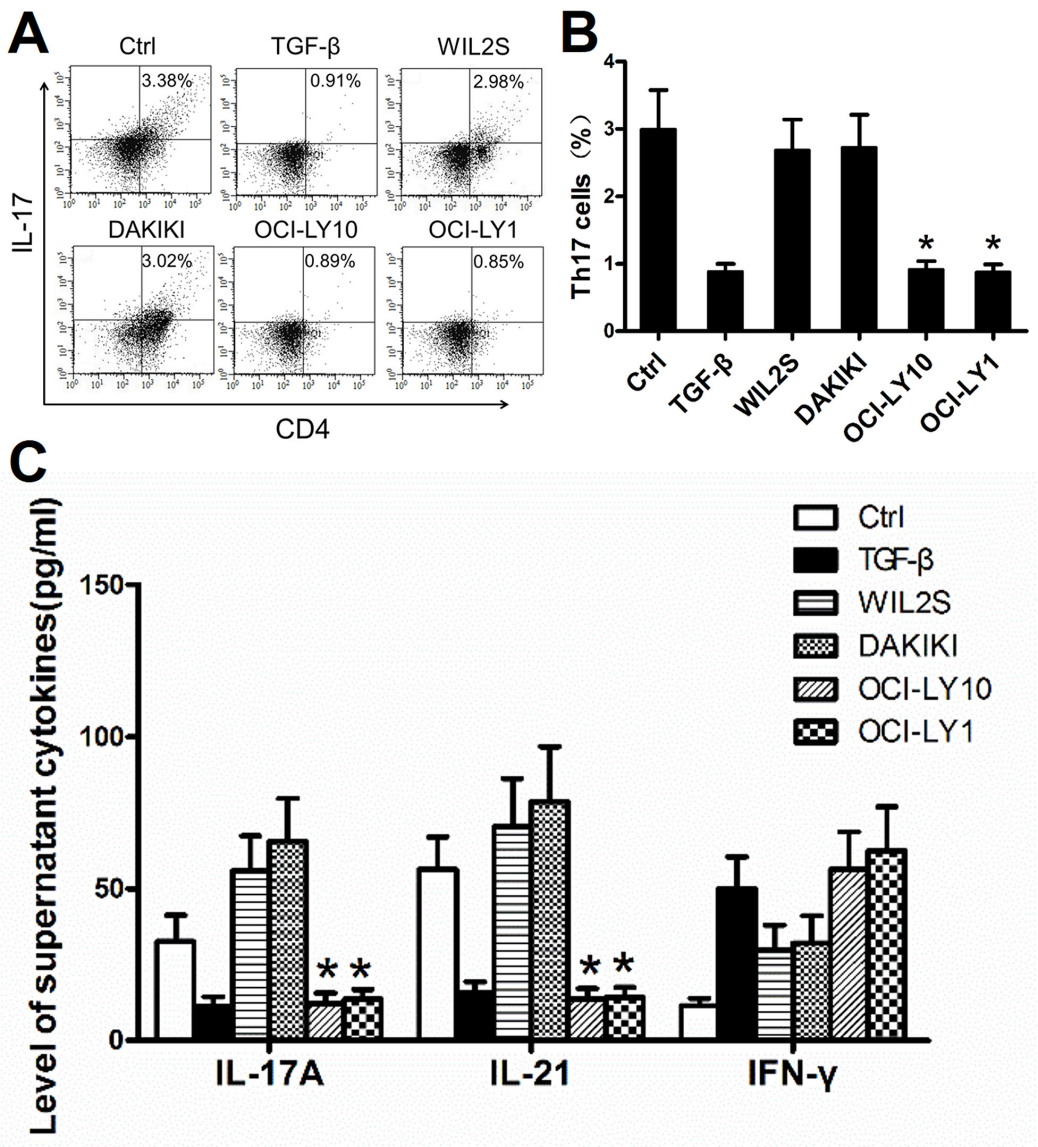
DLBCL cell lines suppressed the generation of Th17 cells and related cytokines *in vitro* **(A)** FACS plots of Th17 cell percentages in PBMCs cultured *in vitro*, Data represent one of three independent experiments. PBMCs from healthy donors were divided into six groups and co-cultured with the DLBCL cell lines OCI-LY10 (ABC subtype) and OCI-LY1 (GCB subtype), human B lymphoblast cell lines WIL2S and DAKIKI, and the cytokine TGF-β. The blank control group only included PBMCs. All six groups were co-cultured with IL-2 for 7 days and then detected by FACS. **(B)** Graph of Th17 cell mean percentages from one of three experiments. **(C)** Mean level of cytokines in the supernatants of the six co-culture groups *in vitro* from one of three experiments. Cytokines were detected by ELISA. Error bars represent SD. Significance was determined using single-factor analysis of variance (one-way ANOVA) Student-Newman-Keulor/Dunnett's T3 test. (*, *P*<0.05 compared with the Ctrl, WIL2S or DAKIKI group).

We found that PBMCs co-cultured with OCI-LY10 and OCI-LY1 had a lower level of Th17 cells *in vitro* than the control, WIL2S, or DAKIKI groups (Figure 3A and 3B, P < 0.05). We repeated these experiments three times.

Secondly, Th17 cell-related cytokine levels were measured in the supernatants of the six co-culture groups by ELISA. IL-17A and IL-21 were expressed at lower levels in the supernatants of the OCI-LY10 and OCI-LY1 groups than in the control, WIL2S, or DAKIKI groups (Figure 3C, *P* < 0.05). IFN-γ expression did not differ significantly between the tumor cell lines and human B lymphoblast cell lines (Figure 3C, *P* > 0.05). These data suggested that the DLBCL cell lines inhibited Th17 cell generation.

### The expression of IRF8 was increased in tumor tissues from DLBCL patients

**Figure 4:**
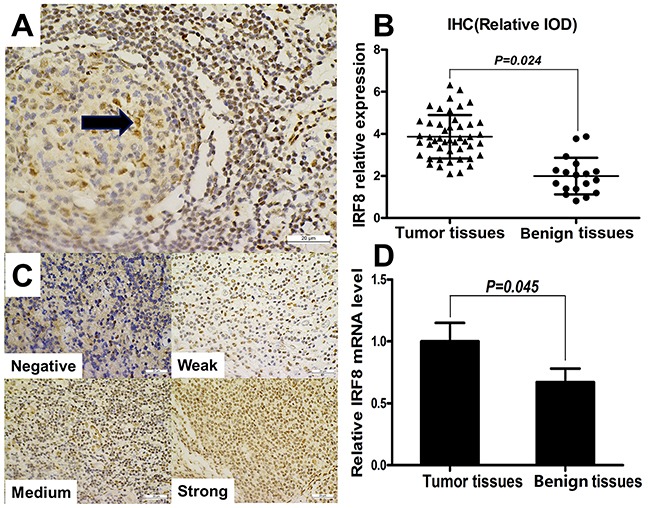
The expression of IRF8 increased in tumor tissues from DLBCL patients **(A)** IRF8 was expressed in the nucleus of lymphocytes in benign lymphoid tissue, and it was highly expressed in the germinal center of lymphoid follicles (×40). **(B)** Graph of IRF8 relative expression (relative IOD) in IHC from tumor tissues (n=48) and benign tissues (n=18). **(C)** Immunohistochemical staining showed various intensities of IRF8 expression in the nucleus of tumor cells or tumor-infiltrating lymphocytes (×40). **(D)** Graph of relative IRF8 transcriptional level in tumor tissues (n=48) and benign tissues (n=18). Error bars represent SD. Significance was determined using independent-sample Student's t/t′ test. The *P* value is indicated in each graph.

To determine whether IRF8 affects Th17 cells generation in the tumor microenvironment, we investigated IRF8 expression in tumor tissues and benign tissues by IHC and quantitative real-time PCR (qPCR), respectively. The relative IOD of IRF8 and IRF8 mRNA levels were analyzed in paraffin-embedded tumor specimens from 48 DLBCL patients and in benign specimens from 18 controls. IRF8 was expressed in the nucleus of lymphocytes in benign lymphoid tissue, and it was highly expressed in the germinal center of lymphoid follicles (Figure 4A). IRF8 was expressed at various levels in the nuclei of tumor cells or TILs (Figure 4C). The relative IOD of IRF8 and the relative IRF8 transcriptional level were significantly higher in tumor tissues than in benign tissues, respectively (Figure 4B, *P* = 0.024; Figure 4D, *P* = 0.045).

### Th17 cell generation was induced by IRF8 knockdown and inhibited by IRF8 overexpression in DLBCL cell lines *in vitro*

**Figure 5:**
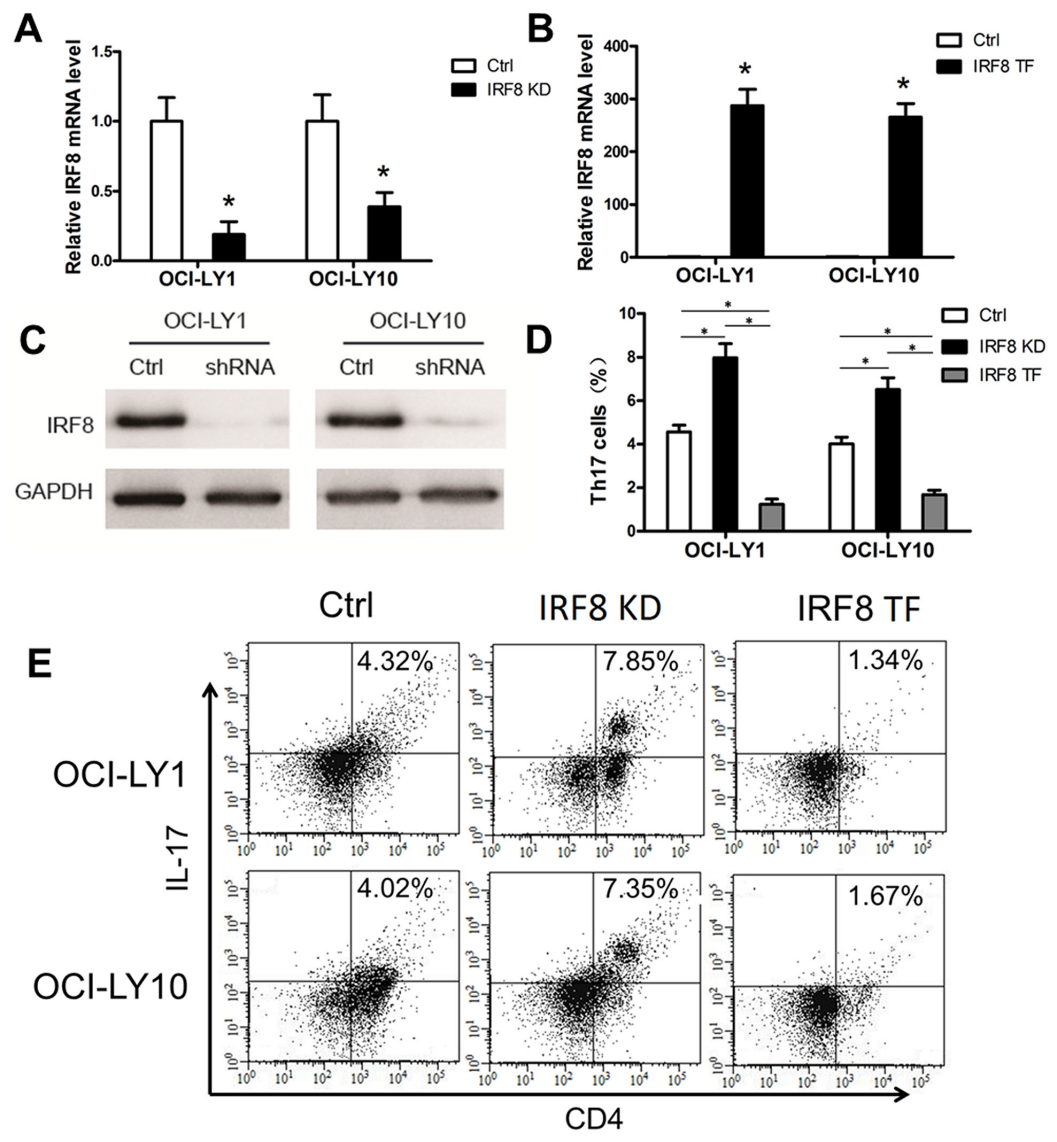
The decline of IRF8 in DLBCL cell lines promoted the generation of Th17 cells and the increase of IRF8 inhibited it *in vitro* One shRNA clone was used to knock down the level of IRF8 in the DLBCL cell lines OCI-LY1 and OCI-LY10. The level of IRF8 transcripts **(A)** and IRF8 proteins **(C)** decreased in OCI-LY1 and OCI-LY10 with IRF8 knockdown (IRF8 KD). **(B)** OCL-LY1 and OCL-LY10 were transfected with lentiviruses encoding IRF8 (IRF8 TF); the transfected cells expressed high levels of IRF8 mRNA. **(D)** The percentage of Th17 cells from the IRF8 KD group was significantly higher than that from the Ctrl group; the IRF8 TF group was significantly lower than the Ctrl group and the IRF8 KD group. **(E)** FACS plots of Th17 cell percentages in PBMCs cultured *in vitro*. Data represent one of three independent experiments. PBMCs from healthy donors were co-cultured with OCI-LY1 and OCI-LY10 (Ctrl), IRF8 KD, or IRF8 TF. All three groups were co-cultured with IL-2, TGF-β and IL-1β for 7 days and then detected by FACS. Error bars represent SD. Significance was determined using independent-sample Student's t/t′ test (two groups) or single-factor analysis of variance (one-way ANOVA) Student-Newman-Keulor/Dunnett's T3 test (three groups). (*, *P*<0.05).

Based on our results showing IRF8 overexpression in the DLBCL tumor microenvironment and previous findings showing that IRF8 inhibits Th17 differentiation in CD4+T cells in mice [26], we investigated whether IRF8 affected the generation of Th17 cells in the tumor microenvironment *in vitro*. Lentiviral-mediated short hairpin RNA (shRNA) transfection was used to silence IRF8 in the DLBCL cell lines OCI-LY1 and OCI-LY10. The results showed that the shRNA significantly downregulated IRF8 at the mRNA and protein levels in OCI-LY1 and OCI-LY10 cells (Figure 5A and 5C).

Then, a lentiviral vector was used to transfect the LentiORF-IRF8 clone into OCI-LY1 and OCI-LY10. The results showed that IRF8 mRNA was significantly increased in transfected OCI-LY1 and OCI-LY10 cells (Figure 5B).

Finally, we performed a co-culture experiment *in vitro* to determine whether IRF8 affects Th17 cell development. PBMCs from healthy donors were co-cultured with IRF8 knockdown (IRF8 KD) or IRF8 transfected (IRF8 TF) OCI-LY1 and OCI-LY10 cells, with untransfected OCI-LY1 and OCI-LY10 as controls. All three groups were co-cultured with IL-2, IL-6 and TGF-β for 7 days and then detected using FACS. We found that Th17 cells percentage from the IRF8 KD group was significantly higher than that from the control (Ctrl) group; the IRF8 TF group was significantly lower than the Ctrl group and the IRF8 KD group. (Figure 5D and 5E, *P* < 0.05). We repeated these experiments three times. These data suggested that knockdown of IRF8 in DLBCL cell lines promoted the generation of Th17 cells and overexpression of IRF8 inhibited it *in vitro*.

### The effect of IRF8 overexpression on inhibiting the generation of Th17 cells was mediated by the downregulation of RORγt

**Figure 6:**
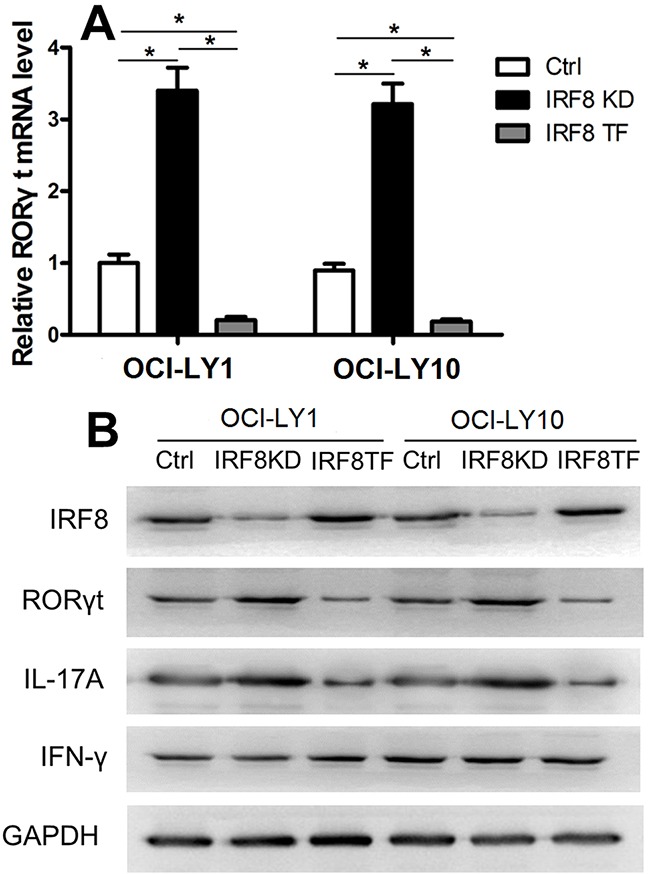
IRF8 suppressed RORγt expression in CD4+T cells **(A)** The RORγt mRNA level was significantly higher in the IRF8 KD group and lower in the IRF8 TF group compared with the Ctrl group. **(B)** Western blot analysis of the Ctrl, IRF8 KD, and IRF8 TF groups in CD4+T cells using specific antibodies against IRF8, RORγt, IL-17, IFN-γ, and *GAPDH*. *GAPDH* was used as a loading control. Error bars represent SD. Significance was determined using single-factor analysis of variance (one-way ANOVA) Student-Newman-Keulor/Dunnett's T3 test. (*, *P*<0.05).

The nuclear hormone receptor RORγt is the crucial transcription factor regulating human Th17 cells differentiation [9–11]. Furthermore, IRF8 was shown to interact with RORγt and suppress the development of Th17 cells in mice [28]. The present study aimed to investigate whether IRF8 in DLBCL cell lines was associated with RORγt and affected its expression in CD4+T cells. We isolated CD4+T cells from the three groups of co-cultures (including the Ctrl group, the IRF8 KD, group and the IRF8 TF group). We detected the transcriptional level of RORγt in CD4+T cells from the Ctrl group, the IRF8 KD group, and the IRF8 TF group by qPCR. The RORγt mRNA level was significantly higher in the IRF8 KD group and lower in the IRF8 TF group compared with that in the Ctrl group (Figure 6A, *P* < 0.05). The levels of IRF8, RORγt, IL-17A, and IFN-γ proteins were analyzed in CD4+T cells from the Ctrl group, the IRF8 KD group, and the IRF8 TF group by western blotting. The protein level of IRF8 was decreased in the IRF8 KD group, while the protein levels of RORγt and IL-17A were decreased in the IRF8 TF group and increased in the IRF8 KD group (Figure 6B). The protein levels of IFN-γ did not differ between the three groups (Figure 6B). These results indicated that IRF8 may suppress the expression of RORγt and IL-17A in CD4+T cells. Therefore, the effect of IRF8 overexpression on inhibiting the generation of Th17 cells may depend on the downregulation of RORγt.

### Increased expression of IRF8 in tumor tissues predicted unfavorable survival for DLBCL patients

**Figure 7:**
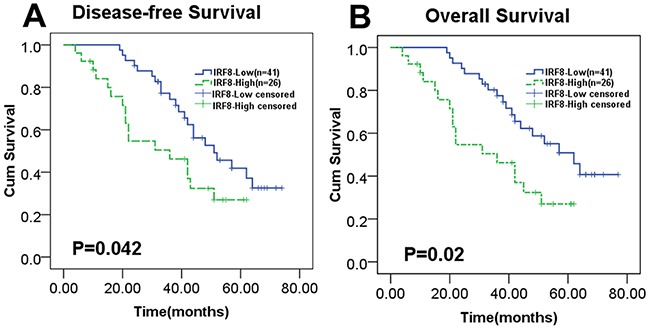
Increased expression of IRF8 in tumor tissues predicted worse DLBCL patient survival A total of 67 DLBCL patients were divided into two groups based on the median relative IOD (3.75) of IRF8 in tumor tissues. **(A)** and **(B)** The disease-free survival and overall survival of DLBCL patients were significantly decreased with increased expression of IRF8 in tumor tissues, as shown in Kaplan-Meier survival analysis plots (*P*=0.042 and *P*=0.02). Significance was determined using the Log Rank test.

It has been reported that the GCB subtype was associated with low levels of IRF8, on the contrary, the non-GCB subtype was associated with high levels of IRF8 [25]. Patients with the GCB subtype generally present favorable survival times compared with the non-GCB subtype [29]. Here, we investigated whether different levels of expression of IRF8 in tumor tissues could affect the survival of patients with DLBCL. We analyzed the expression of IRF8 by IHC in paraffin-embedded tumor specimens from 67 DLBCL patients, of which five showed no IRF8 expression and the others showed various levels of IRF8 expression. DLBCL patients were separated into two groups according to the median relative IOD (3.75) of IRF8 in tumor tissues. The baseline clinical characteristics of the two groups did not differ significantly (Table 2). All patients were treated with the R-CHOP regimen [4, 5]. The high IRF8 level group had significantly worse disease-free survival and overall survival than the low IRF8 group (Figure 7A and 7B, *P* = 0.042 and *P* = 0.02).

**Table 2:** Baseline clinical characteristics of DLBCL patients with different IRF8 expression

Variable	IRF8 in tumor tissues	
	Low (n=41)	High (n=26)
**Age (years)**		
<50	15	10
≥50	26	16
*P* value	0.552	
**Gender**		
Male	25	15
Female	16	11
*P* value	0.485	
**Ann Arbor stage^a^**		
I-II	11	7
III-IV	30	19
*P* value	0.392	
**IPI score^b^**		
1-3	28	19
4-5	13	7
*P* value	0.241	

^a^Ann Arbor stage according to Ann Arbor-Cotswald stage (1989).

^b^IPI score, International Prognostic Index score.

## DISCUSSION

DLBCL is a malignant disease which is highly heterogeneous, it has a complex molecular patho-logy, pathogenesis, and different prognosis. The tumor microenvironment plays a critical role in tumorigenesis. In our study, we examined the mechanisms of DLBCL tumorigenesis to identify new prognostic factors. Our results suggested that Th17 cells percentage was lower in DLBCL tumor tissues than in PBMCs or corresponding adjacent benign tissues; accordingly, the level of IL-17A was also lower in tumor tissues than in benign tissues, and the expression of IFN-γ was the opposite. We also showed that DLBCL cell lines inhibited Th17 cells generation *in vitro*. Increased expression of IRF8 was detected in DLBCL tumor tissues, which predicted worse DLBCL patient survival. Analysis of the underlying mechanism revealed that the effect of IRF8 overexpression on inhibiting the generation of Th17 cells may be associated with the downregulation of RORγt in CD4+ T cells.

Since their identification in 2005, Th17 cells have been extensively investigated in relation to tumorigenesis and tumor progression [30]. The distribution of Th17 cells in the tumor microenvironment is a general characteristic of many cancers [12–15]. Th17 cells percentage was significantly higher in tumor tissues than in PBMCs or normal tissues in nasopharyngeal carcinoma [12] and pancreatic cancer [14]. On the contrary, Th17 cells percentage was lower in tumor tissues than in benign tissues in B cell NHL patients [17]. The percentage of Th17 cells and IL-17 concentration in the peripheral blood from DLBCL patients were significantly lower than in that from healthy donors, and the frequency of Th17 cells in peripheral blood significantly increased in relapsed patients compared with in untreated patients or healthy individuals [18]. In this research, the percentage of Th17 cells significantly decreased in DLBCL tumor tissues compared with in PBMCs or corresponding adjacent benign tissues from DLBCL patients, and the percentage of Th17 cells in PBMCs from DLBCL patients was significantly lower than that from healthy volunteers. These results are in agreement with the conclusions of previous studies and imply that Th17 cells are significantly decreased in DLBCL tumor microenvironment or that these cells migrate from the tumor microenvironment to the peripheral blood. Thus, the mechanism of Th17 cells migrating into the peripheral blood needs to be further investigated [12, 31].

In order to further figure out the role of Th17 cells in DLBCL tumorigenesis, we detected several Th17 cell-related cytokines in DLBCL tumor tissues and benign tissues. Our IHC results showed that the expression of the intracellular cytokine IL-17A was lower, whereas that of IFN-γ was higher in tumor tissues compared with in benign tissues. The percentage of Treg cells did not differ significantly between tumor tissues, corresponding adjacent benign tissues, and PBMCs. These data suggested that Th17 cells secrete other T helper cell cytokines such as IFN-γ, which is in agreement with the findings of previous studies [12, 31]. Producing-IFN-γ Th17 cells (Th1/17 cells) may be antineoplastic in DLBCL by secreting IFN-γ, which has been shown to have an antineoplastic effect [32].

The Th17 cells distribution in tumor micro-environment is associated with cytokines and chemokines released from the tumor microenvironments [12, 33]. In the present study, we firstly showed that IRF8 upregulation in DLBCL cell lines inhibited the generation of Th17 cells *in vitro*. Secondly, IRF8 upregulation was detected in DLBCL tumor tissues. Finally, we showed that DLBCL cell lines with IRF8 knockdown promoted the differentiation of Th17 cells, whereas DLBCL cell lines overexpressing IRF8 had the opposite effect. In summary, our results show that increased expression of IRF8 inhibits the Th17 cells differentiation in the tumor environment of DLBCL patients. However, the mechanism underlying the effect of IRF8 upregulation in DLBCL cells on the regulation of Th17 cells is not clear. It is possible that IRF8 may modulate the expression of surface molecules on the tumor cells or the secretion of soluble factors from these tumors, which then in a tumor-extrinsic manner suppresses RORγt in neighboring T cells and their differentiation into Th17 cells.

Some studies have been showed that lymphoma B cells aberrantly express abundant CD70, CD80, or CD86, and the interaction between CD28-CD80/86 and CD27-CD70 plays a role in the generation of Treg cells [34, 35]. By blocking above-mentioned interactions between lymphoma cells and CD4+T cells, Yang et al. inhibited the generation of Treg cells, which subsequently promotes Th17 cell generation [17]. Therefore, IRF8 may promote DLBCL tumor cell growth, which may inhibit the generation of Th17 cells in DLBCL patients.

The nuclear hormone receptor RORγt is a key transcription factor regulating the differentiation of human Th17 cells [9, 11, 36]. In CD4+T cells, IRF8 interacts with RORγt and suppresses the development of Th17 cells in mice [26]. In the present study, we showed that decreased expression of IRF8 upregulated RORγt, whereas increased expression of IRF8 decreased the level of RORγt.

The differentiation of Th17 cells is suppressed by IRF8 in naïve CD4+T cells derived from Irf8^−/−^ or Lck^−^Cre^+^Irf8^fl/fl^ mice [26, 27], which supports our conclusions. However, Yoshida et al. indicated that IRF8 promotes Th17 cell differentiation in an Irf8^−/−^ mouse model [28]. This contradictory conclusion may be due to different roles of IRF8 in regulating Th17 cells in different diseases or cell types. In addition, our study was based on humans, which differs from the above studies. This suggests that IRF8 plays different roles in Th17 cell development in humans and mice. Recently, Wang et al. showed that in patients with Behcet's disease in the active stage, downregulation of IRF8 by RNA interference leads to the loss of IL-27 inhibition of Th17 cell differentiation, which suggested that IL-27 inhibits Th17 cell development through IRF8 [37]. These studies together with our present results suggest that IRF8 in CD4+T cells in the DLBCL microenvironment downregulate RORγt, leading to the suppression of Th17 cell differentiation.

Some scientists showed that the function of IRF8 in tumorigenesis is cell type dependent. It was reported that IRF8 exerts a pro-apoptotic effect by regulating Fas-mediated apoptosis in sarcoma tumor cells [36]. IRF8 is inversely correlated with colon cancer metastasis [38]. A recent study showed that DNA methylation frequently inhibited IRF8 expression, and aberrant IRF8 expression induces apoptosis dependent on cell cycle G2/M arrest [39]. These conclusions support the anti-tumor effect of IRF8. However, Zhou et al. demonstrated that IRF8 plays an anti-apoptotic role through the regulation of MDM2 and TP53 in GC B cells [40]. It was found that the increased expression of IRF8 induced anti-apoptotic effect in B lymphoma cells [23]. Knockout of IRF8 was shown to suppress tumor growth *in vivo* and DLBCL cell proliferation *in vitro* by inhibiting p38 and ERK activation [25]. The patients with low expression of IRF8 showed a positive correlation with the GCB subtype that predicted a better prognosis in DLBCL [25]. These conclusions are in agreement with a pro-tumor effect of IRF8, and this notion was supported by our present results. In the present study, we found that DLBCL patients with higher levels of IRF8 in the tumor microenvironment had significantly worse disease-free survival and overall survival. This indicated that IRF8 plays an oncogenic role in human DLBCL.

High expression of IRF8 associated with unfavorable survival may be related to the suppression of Th17 cell differentiation in DLBCL patients. Our previous study showed that IL-17 inhibits radiation-triggered apoptosis in B lymphoma cells [41]. Meanwhile, a recent study indicated that IL-17 promotes the growth of NHL tumors in human GC B cells [42]. Therefore, high IRF8 expression associated with unfavorable survival may be related to the suppression of IL-17 expression in the tumor microenvironment. Large scale clinical studies are necessary to evaluate the prognostic significance of IRF8 in DLBCL and to elucidate the oncogenic mechanism of IRF8.

The present study provided novel evidence of decreased generation of Th17 cells and IL-17A and increased expression of IFN-γ and IRF8 in the DLBCL tumor microenvironment. IRF8 upregulation in tumor cells inhibited the generation of Th17 cells *in vitro*, and this may be mediated by the downregulation of RORγt. In addition, we found that a high level of IRF8 in the DLBCL tumor microenvironment was a predictor of poor survival in DLBCL patients. These results provide important evidence of the roles of IRF8 and Th17 cells in DLBCL tumorigenesis and prognosis. Recent studies showed that, in addition to the tumor cells themselves, non-tumor cell components in the lesions of DLBCL patients such as intratumoral Tregs, macrophages, and microvessels, affected the efficacy of the R-CHOP regimen [43]. Therefore, we believe that treatment strategies in the future should address non-tumor cells in the DLBCL microenvironment in addition to tumor cells, which may improve the therapeutic effect.

## MATERIALS AND METHODS

### Human samples

Tumor biopsy tissues and peripheral blood samples were collected from 20 newly diagnosed DLBCL patients at Guangzhou First People's Hospital, Guangzhou Medical University in 2014 and 2015. Tumor tissues included 5 nodal and 15 extranodal tissue samples; the corresponding adjacent benign tissues were collected at the same time from 10 of these extranodal tissues. Tumor tissues and benign tissues were defined by examination of frozen pathologic sections by experienced pathologists. None of the patients received radiotherapy, chemotherapy or immunotherapy. The clinical characteristics of the patients are listed in Table 1. Peripheral blood samples were collected from 20 age-matched healthy volunteers. The tumor tissues and adjacent tissues were resected and cultured in RPMI1640 complete medium (Hyclone, South Logan, UT, USA) with a low dose (20 IU/mL) of IL-2 for 2 h to collect sufficient lymphocytes. These lymphocytes were used to generate tumor infiltrating lymphocytes (TILs) and normal-infiltrating lymphocytes (NILs). PBMCs were isolated from blood samples of DLBCL patients and healthy volunteers using the Ficoll–Hypaque method. PBMCs were cultured in RPMI1640 medium containing 100 U/mL penicillin, 100 U/mL streptomycin, and 10% fetal bovine serum (FBS). A total of 67 paraffin-embedded tumor specimens from DLBCL patients and 18 paraffin-embedded benign lymph node specimens from acute lymphadenitis patients were collected at Guangzhou First People's Hospital, Guangzhou Medical University between 2010 and 2014. Their clinical characteristics are shown in Table 3. Patient survival data were collected through phone calls and clinic visits. Survival time was defined as the period from diagnosis to the last visit, relapse, or death. The study protocol received approval from the Ethics Committee of Guangzhou First People's Hospital (2014-SYL-034). Written informed consent was obtained from all participants or their families.

**Table 3:** Clinical characteristics of 67 DLBCL patients

Characteristics	No. (%)
**Age**	
Median	56
Range	31-74
**Gender**	
Male	40(59.7)
Female	27(40.3)
**Ann Arbor stage^a^**	
I-II	18(26.9)
III-IV	49(73.1)
**IPI score^b^**	
1-3	47(70.1)
4-5	20(29.9)

^a^Ann Arbor stage according to Ann Arbor-Cotswald stage (1989).

^b^IPI score, International Prognostic Index score.

### Cell lines and lymphocytes

The DLBCL cell lines OCI-LY10 (ABC subtype) and OCI-LY1 (GCB subtype) and the human B lymphoblast cell lines WIL2S and DAKIKI were purchased from ATCC (Shanghai, China) and cultured in RPMI1640 medium (Hyclone) containing 10% FBS (Hyclone), 4 mM L-glutamine (Sigma–Aldrich, St. Louis, MO, USA), 100 U/mL of penicillin (Hyclone), and 100 U/mL of streptomycin (Hyclone). HEK-293T cells were purchased from ATCC (Shanghai, China) and cultured in Dulbecco's modified Eagle's medium (Hyclone) supplemented with 10% FBS. All cells were cultured in a humidified chamber at 37°C with an atmosphere of 5% CO_2_. TILs and NILs were isolated from DLBCL tumor tissues and normal tissues, which were cultured in RPMI 1640 medium containing 10% FBS supplemented with 4 mM L-glutamine (Sigma-Aldrich), 1 μM 2-mercaptoethanol (Sigma–Aldrich), and recombinant human IL-2 (300 IU/mL) (Hyclone).

### Flow cytometry and antibodies

TILs, NILs, and PBMCs were analyzed by FACS. Cell density was adjusted to 2 × 10^6^/mL. Cells were stimulated by addition of 50 ng/mL phorbol myristate acetate (PMA), 1 μg/mL ionomycin and 10 μg/mL Brefeldin A (BFA) to the medium for 5 h at 37°C and 5% CO2. Then, anti-human-specific antibodies conjugated with fluorescent molecules were added sequentially to the cells. These human antibodies included anti-CD4, anti-IL-17A, anti-CD25, and anti-FOXP3. These were conjugated with fluorescein isothiocyanate (FITC), phycoerythrin, allophycocyanin, or phycoerythrin-Cy7 (BD Biosciences, San Jose, CA, USA). Stained cells were analyzed on an FC500 flow cytometer (Beckman Coulter, CA, USA). CD4+IL-17A+ cells were defined as Th17 cells, whereas CD4+CD25+FOXP3+ cells were defined as Tregs.

### Immunohistochemistry

The expression of IL-17A, IFN-γ, FOXP3, and IRF8 in the 48 tumor tissues and 18 benign tissues was detected by IHC. The following antibodies were used (all from Abcam, Shanghai, China): polyclonal rabbit anti-human IRF8 antibody (5 μg/mL, ab28696), monoclonal mouse anti-human IL-17A antibody (10 μg/mL, ab189377), monoclonal rabbit anti-human IFN-γ antibody (1:100 dilution, ab133566), and monoclonal mouse anti-human FOXP3 antibody (1:50 dilution, ab22510). Monoclonal mouse anti-human IgG1 (1:200 dilution, ab91353, Abcam) was used as a negative control in this study. Tissues were fixed in 10% neutral formaldehyde, embedded in paraffin, sliced and stained with hematoxylin and eosin. Briefly, the paraffin-embedded tissues were serially cut into 4 μm sections, dewaxed, and rehydrated. Sections were then blocked with peroxide and non-immune animal serum and incubated sequentially with primary antibody, biotin-labeled secondary antibody, and streptomycin anti-biotin peroxidase. Finally, the sections were stained with di-n-butyl adipate (DBA), counterstained with hematoxylin, dehydrated, cleared in xylene, and fixed. The expression of IL-17A, IFN-γ, FOXP3, and IRF8 in paraffin sections was quantified by relative IOD. At least three different images of each paraffin section were acquired at ×40 high power microscopic fields, and the IOD of images and mean IOD of each paraffin section were calculated by experienced pathologists using Image-Pro-Plus 6.0 software (Media Cybernetics, Rockville, MD, USA). The paraffin section with the lowest mean IOD was used as the negative control, and the relative IOD was calculated as the mean IOD/negative control.

### Cell co-culture *in vitro*

PBMCs from healthy donors were cultured in T cell medium containing 10% FBS and 100 IU/mL human IL-2 (Hyclone) at a concentration of 2 × 10^6^ cells/well in a 48-well plate and stimulated with OKT3 (1 μg/mL, Hyclone). The PBMCs were co-cultured with the DLBCL cell lines OCI-LY10 (ABC subtype) and OCI-LY1 (GCB subtype), and the human B lymphoblast cell lines WIL2S and DAKIKI, at a 1:1 ratio or in the presence of cytokine TGF-β (3 ng/mL, Hyclone), and the blank control group which included only PBMCs. All groups were co-cultured with IL-2 (100 IU/mL) for 7 days and then detected by FACS to determine the percentage of Th17 cells. Then, the PBMCs were co-cultured with OCI-LY10 and OCI-LY1, and OCI-LY10 and OCI-LY1 with IRF8 KD, and OCI-LY10 and OCI-LY1 with IRF8 TF, at a 1:1 ratio. All three groups were co-cultured with IL-2 (100 IU/mL), TGF-β (5 mg/mL) and IL-1β (10 mg/mL) for 7 days. The two experiments were repeated three times. CD4^+^ T cells were isolated using magnetic beads (Miltenyi Biotec, Auburn, CA, USA) from three groups of co-cultures (including the Ctrl group, the IRF8 KD group, and the IRF8 TF group) following the manufacturer's instructions. The purity of the isolated CD4+T cells was 99% by flow cytometry.

### ELISA

The supernatants of the different cell co-cultures were assessed for IL-17A, IL-21, and IFN-γ by ELISA following the manufacturer's instructions. The ELISA test was repeated three times. All ELISA kits were purchased from Biolegend (San Diego, CA, USA).

### RNA preparation and quantitative real-time PCR

Total RNA was isolated from cells using the Trizol reagent (Invitrogen, Shanghai, China) according to the manufacturer's instructions. RNA was reverse-transcribed into cDNA using a Thermo Scientific Revert Aid First Strand cDNA Synthesis Kit (Thermo, Shanghai, China) according to the manufacturer's protocols. Quantitative real-time PCR (qPCR) was performed using SYBR Green PCR Master Mix (Roche, Shanghai, China) on a Light Cycler 480II system (Roche). The levels of IRF8 and RORγt were normalized to that of glyceraldehyde-3-phosphate dehydrogenase (*GAPDH*). The primers (5′–3′) used for qPCR included IRF8 for-ward GTAGCATGTATCCAGGACTGATTTG and reverse GCACAGCGTAACCTCGTCTTC, RORγt for-ward TGAGAAGGACAGGGAGCCAA and reverse CCACAGATTTTGCAAGGGATCA, and *GAPDH* forward GCACCGTCAAGGCTGAGAAC and reverse TGGTGAAGACGCCAGTGGA.

### Western blot analysis

Cells were lysed using sodium dodecyl sulfate (SDS) buffer containing proteinase inhibitors (Roche). Equal amounts of protein (50 μg) were separated by 10% SDS-polyacrylamide gel electrophoresis (SDS-PAGE), and transferred onto PVDF membranes (Bio-Rad, Shanghai, China). The membranes were blocked and incubated with specific antibodies overnight at 4°C. Antibodies against GAPDH, RORγt, IL-17A, IFN-γ, and IRF8 were purchased from Santa Cruz Biotechnology (Dallas, TX, USA). Then, membranes were incubated with horseradish peroxidase-labeled secondary antibody (Santa Cruz Biotechnology). The protein bands were visualized using an enhanced chemiluminescence reagent.

### Knockdown of IRF8 expression in DLBCL cell lines

IRF8-specific shRNA were delivered by lentiviral infection to knock down IRF8 expression in DLBCL cell lines. The RNAi Consortium (TRC) human IRF8 shRNA and Trans-Lentiviral shRNA Packaging Kit with Calcium Phosphate Transfection Reagent (including pGIPZ™ Non-silencing Control Vector DNA and HEK293 cells) were purchased from Dharmacon (Lafayette, CO, USA). The IRF8 shRNA sequence was TTCTTTAATCATGATGCGGGC. The IRF8-specific shRNA was cloned into pLKO.1 puro plasmids. Briefly, as described previously [25, 26], HEK293T cells were seeded at a density of 5.5 × 106 cells/14 mL in a 100 mm plate. The next day, 42 μg shRNA plasmids was transfected into HEK293T cells together with 30 μL of the Trans-Lentiviral Packaging Kit (including five plasmids: pTLA1-PAK, pTLA1-ENZ, pTLA1-ENV, pTLA1-TOFF, and pTLA1-TAT/REV) using calcium phosphate (105 μL). At 48 h after transfection, the supernatant containing lentiviral particles was harvested and filtered through a 0.45 μm-diameter filter, and used to infect DLBCL cell lines. Puromycin was used to select cells. The levels of IRF8 mRNA and protein were evaluated by qPCR and western blotting.

### IRF8 transfection into DLBCL cell lines

Lentiviral transfection of the LentiORF-IRF8 clone into DLBCL cell lines was used to upregulate IRF8 expression. The CCSB-Broad LentiORF-IRF8 clone and Trans-Lentiviral shRNA Packaging Kit with Calcium Phosphate Transfection Reagent (including Precision LentiORF RFP Control DNA and HEK293 cells) were purchased from Dharmacon. The sequences for the IRF8 ORF were as follows: forward, 5′–CGCAAATGGGCGGTAGGCGTG–3′ and reverse 5′–TACGGGAAGCAATAGCATGA–3′. The ORFs were cloned into pLX304-Blast-V5 vectors. ORF plasmid transfection was performed using procedures similar to those described for shRNA transfection.

### Statistical analysis

All analyses were performed using SPSS 17.0. Numerical data were presented as the mean ± standard deviation. Two-tailed independent-sample Student's t/t′ test were used for comparisons between two groups. Single-factor analysis of variance (one-way ANOVA), Student-Newman-Keulor/Dunnett's T3 test (between each two groups) were used for comparisons among multiple groups. Survival was estimated using the Kaplan-Meier method and the log-rank test. *P* values of <0.05 were considered significant.

## Abbreviations

DLBCL: diffuse large B cell lymphoma
FACS: fluorescence-activated cell sorting
IHC: immunohistochemistry
IL-17: interleukin-17
IOD: integrated optical density
IPI: international prognostic index
IRF8: interferon regulatory factor 8
IRF8 KD: IRF8 knockdown
IRF8 TF: IRF8 transfected
NHL: non-Hodgkin's lymphoma
NILs: normal-infiltrating lymphocytes
PBMCs: peripheral blood mononuclear cells
ROR-γt: retinoid-related orphan receptor γ-t
TILs: tumor-infiltrating lymphocytes
